# Insecticidal potential of *Areca catechu* nut extract against multiple life stages of *Aedes aegypti* and *Aedes albopictus*

**DOI:** 10.1371/journal.pone.0341897

**Published:** 2026-02-02

**Authors:** Zainab Rahman, Madhuri Bharathithasan, Lau Yee Ling, Olawale Quazim Junaid, Intan H. Ishak, Rajiv Ravi

**Affiliations:** 1 Department of Parasitology, Faculty of Medicine, Universiti Malaya, Kuala Lumpur, Malaysia; 2 Faculty of Integrated Life Science, School of Applied Sciences, Quest International University, Ipoh, Perak, Malaysia; 3 Insecticide Resistance Research Group (IRRG), School of Biological Sciences, Universiti Sains Malaysia, Minden, Penang, Malaysia; National Research Centre, EGYPT

## Abstract

This study investigates the insecticidal efficacy of *Areca catechu* nut extract against *Aedes aegypti* and *Aedes albopictus*, in response to increasing insecticide resistance, declining effectiveness of conventional agents, and environmental safety concerns. The primary objectives were to evaluate the adulticidal, ovicidal, and oviposition deterrent activities of methanolic *A. catechu* nut extract across a concentration range of 300–2000 ppm, and to identify its major bioactive constituents. Laboratory bioassays were conducted following World Health Organization protocols. The extract induced dose-dependent adult mortality, with LC_50_ values of 767.501 ppm for *Ae. aegypti* and 758.278 ppm for *Ae. albopictus*. Ovicidal assays showed progressive increases in egg mortality, reaching 100% at 1600 ppm for *Ae. aegypti* and 1400 ppm for *Ae. albopictus*. In oviposition deterrent tests, complete inhibition of egg-laying occurred at concentrations of 900 ppm and above under both dual-choice and non-choice conditions. Observational data confirmed strong repellence, as gravid females avoided treated substrates even in the absence of alternatives. Liquid chromatography–mass spectrometry analysis identified arecoline, arecaidine, and N-lauryldiethanolamine as key constituents with known inhibitory effects on neural and detoxification enzymes in insects. This research provides a comprehensive assessment of *A. catechu* nut extract across multiple mosquito life stages and behavioural endpoints, demonstrating its broad-spectrum efficacy. The results support its potential as a sustainable, plant-derived bioinsecticide for integrated vector control programs targeting *Aedes* mosquitoes and associated disease transmission.

## Introduction

Mosquitoes serve as vectors for pathogens responsible for several widespread human diseases, including malaria, chikungunya, yellow fever, Zika, West Nile virus and dengue [1]. Within the genus *Aedes*, certain species are particularly significant for their role in transmitting arboviruses that cause dengue, chikungunya, yellow fever, and Zika in humans. Among these, *Ae. aegypti and Ae. albopictus* have garnered special attention, as they are identified as primary vectors in dengue transmission [2]. *Ae. aegypti* and *Ae. albopictus* are both highly adapted to urban environments, and their ability to thrive in such settings makes vector control particularly challenging [3,4]. Dengue virus (DENV) is predominantly distributed in urban and semi-urban settings within tropical and subtropical regions, posing a risk to nearly 50% of the global population. Annual estimates indicate between 100 and 400 million infections worldwide. In 2023, over five million dengue cases were reported across more than 80 countries and territories [5]. This increasing trend emphasizes the need for proper and effective vector control management.

Over the past century, mosquito control has primarily relied on neurotoxic chemical insecticides, including pyrethroids, neonicotinoids, chlorinated hydrocarbons, carbamates, and organophosphates. However, the continued and widespread use of these compounds has led to the development of insecticide resistance [6]. The growing resistance of mosquito vectors to insecticides, coupled with the slow progress in developing new drugs and vaccines, has significantly hindered control efforts. Ineffective vector control strategies, the spread of invasive mosquito species, and increased human–vector contact have all contributed to the continuous re-emergence of arboviral diseases. As a result, mosquito control programs are confronted with substantial and rapidly evolving challenges, underscoring the need for novel strategies in disease detection and control as an emerging priority in public health [1]. To overcome these challenges, increasing attention is being directed toward bio-insecticides and biocontrol strategies as environmentally benign and ecologically sound alternatives to conventional chemical pesticides. Derived from natural sources such as plants, bacteria, and fungi, biopesticides present a promising approach for the effective management of invasive pest species while supporting the preservation of ecological integrity [7]. Numerous studies have demonstrated the effectiveness of plant-based formulations against mosquito larvae and adults.

Previous study has demonstrated the successful synthesis of zinc oxide nanoparticles (ZnO NPs) using *Ficus racemosa* L. leaf extract, with notable larvicidal activity against *Ae. albopictus* larvae. High mortality was observed at a concentration of 250 μg/mL, indicating strong susceptibility. The concentration-dependent inhibition of acetylcholinesterase (AChE) and carboxylesterase phosphatase activities suggests a potential mechanism underlying the larvicidal effect. [8]. Another study investigated the larvicidal efficacy of extract from common seaweeds, including *Padina gymnospora*, *P. pavonica*, *Gracilaria crassa*, *Amphiroa fragilissima*, and *Spatoglossum marginatum*. The ethyl acetate extract of *P. gymnospora* exhibited the highest larvicidal activity against *Ae. aegypti*, with an LC₅₀ of 27.0 μg/mL. In comparison, selected extract from *P. gymnospora*, *P. pavonica*, and *A. fragilissima* also demonstrated strong larvicidal activity against *Anopheles stephensi* and *Culex quinquefasciatus*, with LC₅₀ values in a similar range to those observed for *Ae. aegypti* [9]. Collectively, these studies highlight the growing interest and potential of naturally derived substances in mosquito vector control. In this context, the insecticidal potential of *A. catechu* nut extract has gained attention due to previous reports of its larvicidal activity against mosquito vectors, positioning it as a potential botanical resource for further investigation in vector control strategies [10]

*A. catechu* nuts are rich in bioactive constituents such as alkaloids, polyphenols, polysaccharides, and fatty acids, which have been shown to exhibit diverse pharmacological activities, including antibacterial, anthelmintic, antiviral, antioxidant, anti-inflammatory, and antitumor effects. These compounds are also known to influence various physiological systems, particularly the nervous, digestive, and endocrine systems, further emphasizing the broad-spectrum bioactivity and therapeutic potential of *A. catechu* [11]. In addition to their diverse pharmacological activities, *A. catechu* nut extract has recently garnered interest due to their rich phytochemical composition, including alkaloids such as arecoline and arecaidine, which are known to interfere with neural and enzymatic pathways in insects. Previous work [12] demonstrated that the methanolic extract of *A. catechu* nut (MEAN), along with its alkaloid constituents arecoline and arecaidine, demonstrated significant inhibitory effects on key detoxifying enzymes in *Plutella xylostella* larvae. These compounds markedly reduced acetylcholinesterase (AChE) activity, indicating potential disruption of neural transmission. Additionally, arecoline and arecaidine exerted strong inhibitory effects on carboxylesterase (CarE) activity, which plays a crucial role in metabolic detoxification processes in insects. These enzyme inhibition patterns suggest that both the crude extract and its active compounds possess potent insecticidal properties, supporting their potential application as eco-friendly botanical pesticides for the control of *P. xylostella* and other insect pests.

In support of this, previous investigations by our colleagues [10], demonstrated the larvicidal efficacy of *A. catechu* nut extract against early fourth instar larvae of *Ae. aegypti* and *Ae. albopictus* across a concentration range of 200–1600 mg/L. The study reported LC₅₀ and LC₉₅ values of 621 mg/L and 2264 mg/L for *Ae. aegypti*, and 636 mg/L and 2268 mg/L for *Ae. albopictus*, respectively, indicating strong larvicidal toxicity. Importantly, no mortality was observed in non-target organisms, suggesting a favourable environmental safety profile. However, a notable gap in the existing literature is the limited evaluation of *A. catechu* nut extract specifically against *Ae. albopictus*, despite its growing significance as a disease vector. Nevertheless, this study [10] addresses the gap by demonstrating the strong larvicidal activity of the extract against *Ae. albopictus*, supported by comprehensive chemical profiling and bioassay validation, in which gas chromatography–mass spectrometry (GC–MS) analysis of the extract identified several bioactive compounds, including arecaidine, dodecanoic acid, methyl tetradecanoate, tetradecanoic acid, and *n*-hexadecanoic acid. These constituents are widely recognized for their roles in pesticidal, insect-repellent, and insecticidal applications, further supporting the potential of *A. catechu* as a promising botanical resource for mosquito vector control.

Building upon these findings, the current study aims to comprehensively evaluate the insecticidal potential of *A. catechu* nut extract beyond the larval stage. Specifically, this study investigates its adulticidal, ovicidal, and oviposition deterrent activities against *Ae. aegypti* and *Ae. albopictus*, thereby assessing its holistic utility as a natural vector control agent. The results are expected to reinforce the role of *A. catechu* as a sustainable, plant-based alternative to synthetic insecticides in integrated mosquito management programs.

## Methodology

### Soxhlet extraction

*A. catechu* nuts were procured from a local betel nut supplier in Ipoh, Perak, Malaysia. The collection site is geographically located at a latitude of 4.71623 and a longitude of 101.12118. During procurement, areca nuts were identified based on morphological characteristics and comparison with published literature. The nuts were dark brown in colour, exhibiting a short, rounded conical shape approximately one inch in height, with a veined surface resembling that of nutmeg and a small conical embryo at the base [13]. Upon procurement, the nuts were washed thoroughly under running tap water to remove surface debris, then dried in a hot-air oven at 40 °C for 24 hours. The dried material was ground into a fine powder using an industrial grinder and stored in an airtight vacuum container until further use.

For extraction, 40g of powdered *A. catechu* nuts were subjected to Soxhlet extraction using 1000 mL of absolute methanol, following methods outlined by previous studies [14,15]. Methanol was selected as the extraction solvent due to its high efficiency in extracting tannins and total phenolic compounds, both of which are present in *A. catechu* seeds [16]. Additionally, due to its strong solvating ability for both polar and moderately non-polar compounds [17], methanol is particularly suitable for the extraction of alkaloids such as arecoline and arecaidine. Furthermore, its low viscosity [18] promotes efficient mass transfer and diffusion during Soxhlet extraction. Following extraction, methanol was removed using a rotary evaporator at 45 °C, yielding 4 gm of crude extract, which was stored at 4 °C for subsequent experimental analysis.

### *Aedes* mosquito rearing

*Ae. aegypti* and *Ae. albopictus* eggs were obtained from Vector Control Research Unit (VCRU) at Universiti Sains Malaysia (USM), Penang, Malaysia. These eggs were hatched in controlled laboratory conditions for 24 hours in de-chlorinated water at 24–28 °C, pH 6.0–7.0, relative humidity of 75 ± 5% and dissolved oxygen of 5.4–6.0 mg/L. Larvae were reared on a diet composed of dog biscuit, sun-dried beef liver, yeast, and milk powder in a ratio of 2:1:1:1, ground into a powdered form, and maintained on this diet until pupation [19,20]. Thereafter, pupae were transferred into cages allowing them to emerge into adults. The cages were provided with sucrose solution to ensure food source for the mosquitoes.

### Adulticidal bioassays

**Fig 1:**
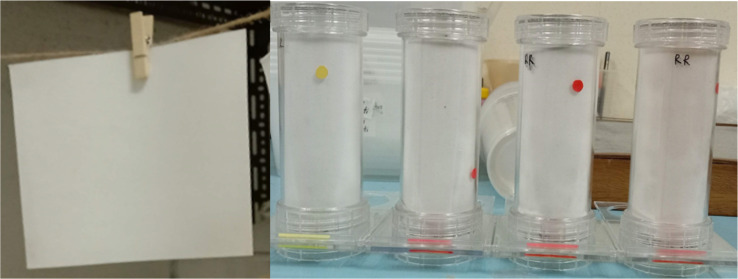
Preparation and application of treated filter papers for adulticidal bioassays using *A. catechu* nut extract.

Adulticidal bioassays and their replicates were conducted in accordance with the standard protocols established by the World Health Organization [21], supplemented by methodologies previously employed by our research group [22]. Serial dilutions of *A. catechu* crude extract were prepared using methanol, producing a concentration range of 600–2000 ppm. The prepared solutions were uniformly applied to filter papers (140 × 120 mm), as illustrated in Fig 1. Control treatments consisted of filter papers of identical dimensions impregnated solely with methanol. All impregnated filter papers were air-dried at room temperature for 24 hours to ensure complete evaporation of the solvent prior to use in bioassays. Female *Ae. aegypti* and *Ae. albopictus* mosquitoes, aged 3–5 days, were selected for testing due to their reproductive maturity and consistent physiological responses at this age.

For each treatment, 25 female mosquitoes were aspirated and placed in WHO-standard holding tubes. The mosquitoes were acclimatized for 1 hour under laboratory conditions, during which any weak, injured, or deceased individuals were replaced to ensure uniformity across test groups. Following acclimatization, filter papers impregnated with the methanolic extract were placed into the exposure tubes of the WHO bioassay kits, after which the mosquitoes were gently transferred from the holding tubes to the corresponding exposure chambers. The exposure period lasted for 60 minutes, during which knockdown was recorded at 5-minute intervals. Mosquitoes were considered knocked down if they were unable to stand or initiate flight. At the conclusion of the exposure period, all mosquitoes were transferred into clean paper cups provided with a 10% sucrose solution enriched with vitamin B complex via soaked cotton pads. The mosquitoes were maintained for a 24-hour recovery period under standard insectary conditions. Mortality was recorded at the end of this period. A parallel control group, exposed to methanol-impregnated papers, was included in all assays, with no mortality observed. Each treatment and control group were replicated four times for both mosquito species to ensure reproducibility and statistical reliability.

### Ovicidal bioassays

A total of 100 eggs were collected from each mosquito species, *Ae. aegypti* and *Ae. albopictus*. Various test concentrations of *A. catechu* nut extract (300 ppm, 600 ppm, 900 ppm, 1200 ppm, 1400 ppm, 1600 ppm, 1800 ppm, and 2000 ppm) were prepared using methanol as the solvent. Distilled water mixed with 10% methanol served as the control and was placed in separate cups. Each concentration, including the control, was tested in five replicates to ensure the reliability of results. Following treatment, eggs from each group were examined under a microscope and subsequently transferred to cups containing distilled water for the evaluation of hatching success. Egg mortality was assessed based on the number of unhatched eggs with unopened opercula [23,24]. After 48 hours of exposure, hatching rates were evaluated, and egg mortality was calculated using the formula [24]:


Egg mortality (%)=(Number of hatched larvae / Total number of eggs in treated water)×100

### Oviposition deterrence test

The dual-choice oviposition bioassay was conducted to determine the deterrent activity of *A. catechu* nut extract on gravid females of *Ae. aegypti* and *Ae. albopictus* [22]. A total of fifteen gravid female mosquitoes, aged five days, from each species were released into an insect cage measuring 30 cm × 30 cm × 30 cm under controlled room conditions. Throughout the assay, the mosquitoes were provided with continuous access to a 10% sucrose solution. The experiment was performed in both choice and non-choice formats, with experimental set-up for each treatment concentration replicated five times.

In the dual-choice test, two 50 mL plastic cups containing dechlorinated water mixed with 10 percent methanol served as controls, while two additional cups of the same volume were filled with *A. catechu* nut extract dissolved in methanol at varying concentrations ranging from 300 ppm to 1800 ppm. To facilitate oviposition, a strip of Whatman No. 1 filter paper was placed inside each cup, with the lower half submerged in the solution and the upper half exposed above the liquid surface, providing an appropriate site for egg-laying. The treated and control cups were placed diagonally opposite each other in the cage in each replicate to minimise positional bias in oviposition behaviour. Each concentration in the non-choice test was also replicated five times.

For the non-choice test, a single 50 mL plastic cup containing *A. catechu* nut extract at the test concentration and prepared with filter paper in the same manner was placed in the centre of a separate cage. Fifteen gravid female mosquitoes were introduced into each cage, with continuous access to 10 percent sucrose solution. Each concentration in the non-choice test was also replicated three times. No control cup was included in this setup, and oviposition activity was assessed solely based on the number of eggs laid in the treated cup.

After three days of exposure, all filter papers were collected, and the number of eggs deposited was counted using a stereomicroscope. The effective repellency percentage (ER) for each concentration was calculated using the following formula [22]:


$$\text{ER}=\frac{\left(\text{NC}-\text{NT}\right)}{\text{NC}}\text{x}\;\text{100}\%$$

where ER = percent effective repellency; NC = number of eggs in control; and NT = number of eggs in treatment.

Also, oviposition activity index (OAI) for dual-choice test was calculated using the formula [25]:


$$\text{OAI}=\frac{\text{NT}-\text{NC}}{\text{NT}+\text{NC}}$$

where NT = number of eggs in the treated substrate cups and NC = number of eggs in the control substrate cups.

### Liquid chromatography-mass spectrometry (LCMS)

LC–MS analysis was conducted using a Shimadzu LCMS system equipped with an electrospray ionization (ESI) source operating in both positive and negative ion modes. Chromatographic separation was achieved on an Agilent TC-C18(2) reversed-phase column (2.0 × 150 mm, I.D., 5 µm particle size) maintained at 25°C. The mobile phase consisted of MeCN/H₂O/HCOOH in a ratio of 3:92:5 (v/v/v), delivered isocratically at a flow rate of 0.25 mL/min. The injection volume was 5 µL. Mass spectrometric detection was performed in full scan mode over a mass range of m/z 40–1300, covering the anticipated empirical range of polar compounds. The interface, desolvation line, and heat block temperatures were set to 250°C, 250°C, and 400°C, respectively. Nitrogen was used as both nebulising and drying gas. Data acquisition and processing were carried out using Shimadzu LabSolutions software. Samples were filtered through a 0.22 µm PTFE membrane filter prior to analysis to ensure compatibility and prevent column clogging.

## Results

### Adulticidal bioassay

**Fig 2:**
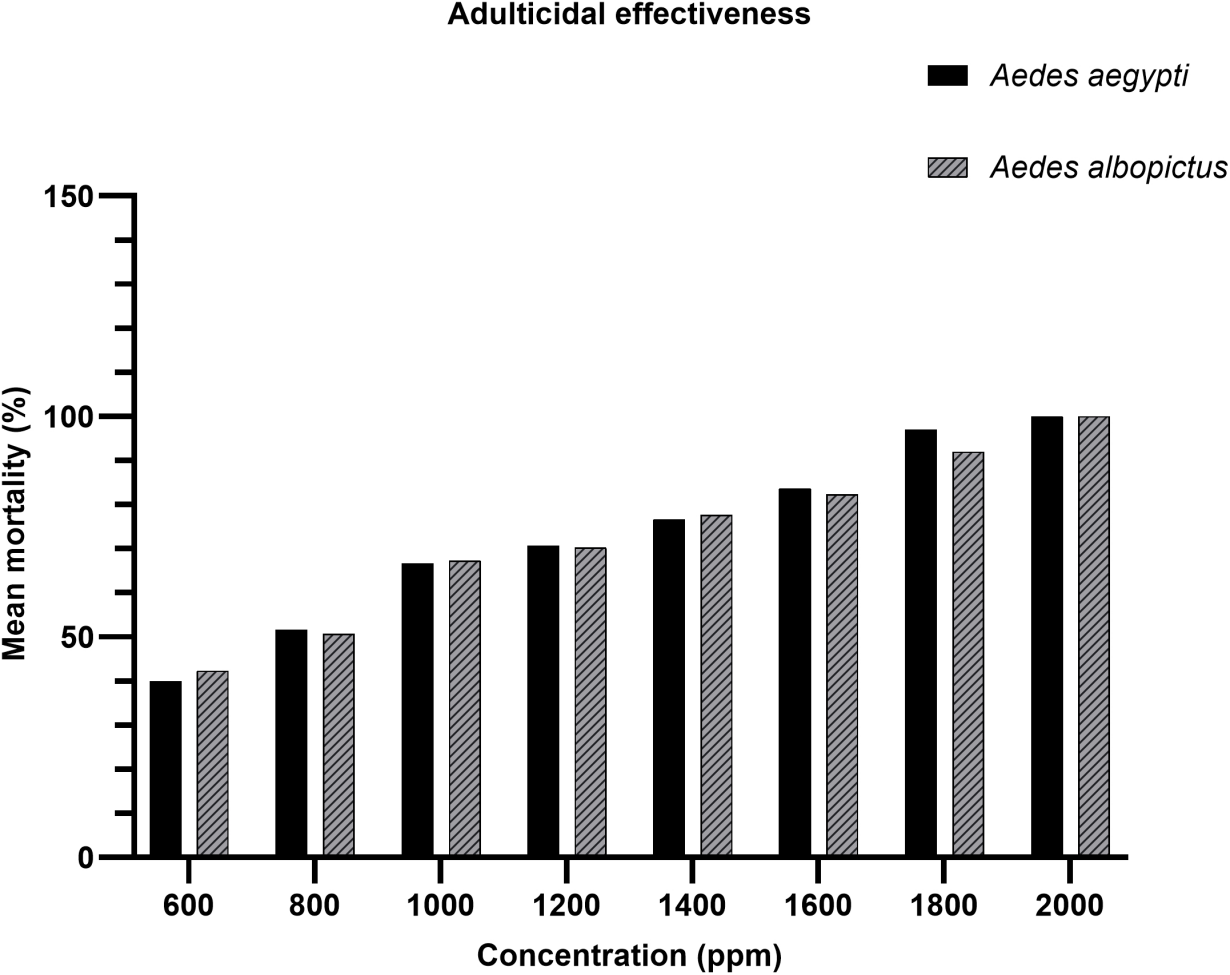
Dose-dependent adulticidal activity of *A. catechu nut* extract against both *Aedes* species.

The adulticidal bioassay revealed a concentration-dependent increase in mosquito mortality for both *Ae. aegypti* and *Ae. albopictus* following exposure to *A. catechu* nut extract. As illustrated in Fig 2, the mortality rates increased progressively with increasing concentrations, beginning around 40–45% at 600 ppm and reaching 100% at 2000 ppm for both species. Probit regression analysis supported these trends. For *Ae. aegypti*, the LC₅₀ and LC₉₅ values were 767.501 ppm and 2091.219 ppm as shown in Table 1. Similarly, *Ae. albopictus* exhibited LC₅₀ and LC₉₅ values of 758.278 ppm and 2271.995 ppm, respectively (Table 2).

**Table 1:** Adulticidal efficacy of *A. catechu* nut extract against *Ae. aegypti.*

Chi-sq. test	Sig^a^	95% Confidence limits					
		LC_50_	Lower limit of LC_50_	Upper limit of LC_50_	LC_95_	Lower limit of LC_95_	Upper limit of LC_95_
18.522	0.005	767.501	593.684	893.869	2091.219	1692.187	3157.680
**Regression equation**		Y = −10.901 + 3.778x					

LC_50_: lethal concentration for 50% mortality after 24 hours in ppm; LC_95_: lethal concentration for 95% mortality after 24 hours in ppm. ^a^Since the significance level is less than 0.050, a heterogeneity factor was used in the calculation of confidence limits.

**Table 2:** Adulticidal efficacy of *A. catechu* nut extract against *Ae. albopictus.*

Chi-sq. test	Sig^b^	95% Confidence limits					
		LC_50_	Lower limit of LC_50_	Upper limit of LC_50_	LC_95_	Lower limit of LC_95_	Upper limit of LC_95_
14.672	0.023	758.278	591.216	881.070	2271.995	1826.946	3427.061
**Regression equation**		Y = – 9.943 + 3.453x					

LC_50_: lethal concentration for 50% mortality after 24 hours in ppm; LC_95_: lethal concentration for 95% mortality after 24 hours in ppm. ^b^Since the significance level is less than 0.050, a heterogeneity factor was used in the calculation of confidence limits.

Fig 2 illustrates the adulticidal activity of the tested extract against *Ae. aegypti* and *Ae. albopictus* across a concentration range of 600–2000 ppm. A clear dose-dependent increase in mean mortality (%) was observed for both species. At the lowest concentration (600 ppm), *Ae. aegypti* exhibited 40% mortality, while *Ae. albopictus* showed a slightly higher mortality rate of 42%. Mortality rates increased progressively with concentration, with a more pronounced escalation occurring beyond 1000 ppm, indicating a potential toxicity threshold near the LC₅₀. Near-complete (100%) mortality was achieved at 2000 ppm for both species, confirming the extract’s strong adulticidal efficacy. While both species demonstrated similar trends in susceptibility, *Ae. albopictus* exhibited marginally higher mortality across most concentrations, suggesting slightly greater sensitivity to the extract compared to *Ae. aegypti*. Control groups treated with solvent (methanol) alone recorded 0% mortality, confirming the bioactivity of the extract as the sole cause of observed effects. Thus, these findings indicate that *A. catechu* nut extract possesses strong adulticidal properties against both *Aedes* species

### Ovicidal bioassay

**Fig 3:**
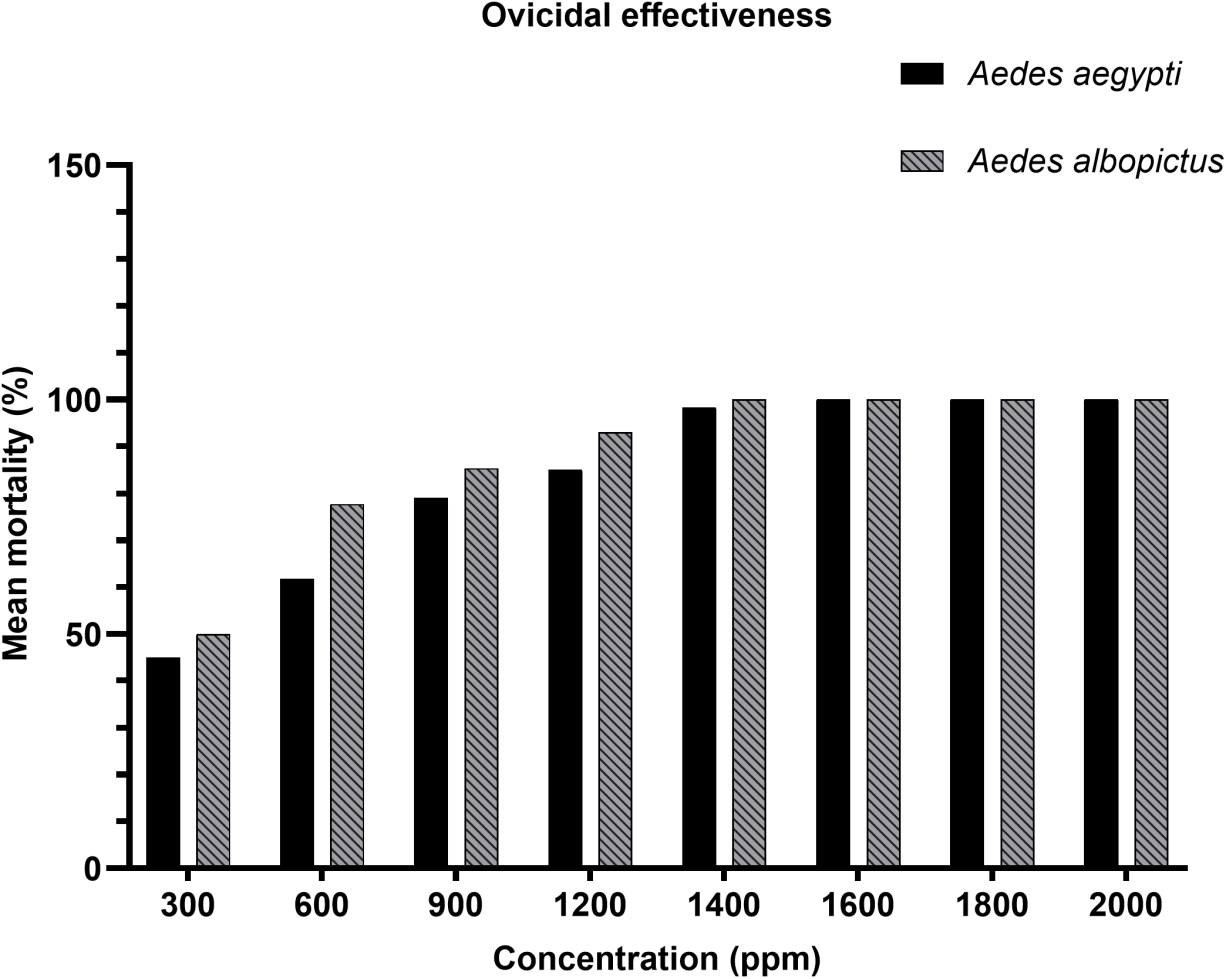
Ovicidal effectiveness of *A. catechu* nut extract against *Ae. aegypti* and *Ae. albopictus.*

Following the adulticidal assessments, the ovicidal efficacy of *A. catechu* nut extract was evaluated by assessing egg mortality based on hatching success. A concentration-dependent reduction in hatchability was observed in both *Ae. aegypti* and *Ae. albopictus*. As presented in Table 3, *Ae. aegypti* exhibited 45% egg mortality at 300 ppm, which increased to 62% at 600 ppm, 79% at 900 ppm, and 85% at 1200 ppm. Complete mortality was achieved at 1600 ppm and maintained at all higher concentrations. For *Ae. albopictus*, egg mortality was slightly higher across all concentrations, beginning at 50% at 300 ppm and rising to 78% at 600 ppm, 85% at 900 ppm, and 93% at 1200 ppm. Full egg mortality (100%) was reached at 1400 ppm and sustained at all subsequent concentrations (Table 3). In contrast, 0% mortality was observed in both species under control conditions, indicating that the observed effects were solely attributable to the extract. This dose-response relationship is clearly illustrated in Fig 3, where at 300 ppm, *Ae. albopictus* exhibited slightly higher mortality (50%) than *Ae. aegypti* (45%). Both species showed a marked increase in mortality between 600 and 1200 ppm, followed by a plateau from 1400 ppm onwards, where near-total egg mortality was achieved. These results indicate a dose-dependent response, with *Ae. albopictus* demonstrating marginally greater sensitivity at lower concentrations.

**Table 3:** Egg mortality (mean %) in *Aedes* species exposed to varying concentrations of *A. catechu* nut extract.

Mosquitoes	Control	Concentrations (ppm)							
		300	600	900	1200	1400	1600	1800	2000
***Ae. albopictus***	0	50	78	85	93	100	100	100	100
***Ae. aegypti***	0	45	62	79	85	98	100	100	100

Values indicate the percentage of unhatched eggs 48 hours post- exposure to different concentrations of methanolic *A. catechu nut* extract.

### Oviposition deterrence test

**Fig 4:**
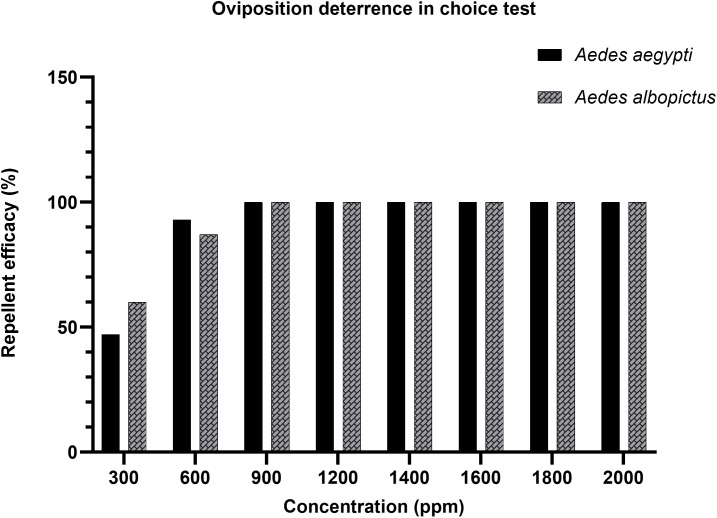
Effective repellency (mean%) in *Ae. aegypti* and *Ae. albopictus* under choice test conditions.

Dual-choice and non-choice oviposition assays were conducted to assess the repellency potential of *A. catechu* nut extract against gravid females of *Ae. aegypti* and *Ae. albopictus*. A clear concentration-dependent oviposition deterrent effect was observed across all tested concentrations. In the dual-choice assay (Table 4), repellency was lowest at 300 ppm, with *Ae. aegypti* and *Ae. albopictus* showing 47% and 60% deterrence, respectively. Repellency increased markedly at 600 ppm, reaching 93% for *Ae. aegypti* and 87% for *Ae. albopictus*. All higher concentrations (≥900 ppm) resulted in complete oviposition deterrence (100%) for both species. These percentage-based trends were consistently reflected in the oviposition activity index (OAI) values, which became progressively more negative with increasing concentration, indicating stronger oviposition deterrence. Moderate deterrence observed at 300 ppm corresponded to moderately negative OAI values, whereas higher concentrations associated with high or complete repellency yielded strongly negative OAI values approaching −1.00. At concentrations producing 100% deterrence, OAI values reached −1.00 across all replicates, confirming complete avoidance of treated oviposition substrates. These findings are illustrated in Fig 4, which shows marked increase in repellence between 300 ppm and 600 ppm, with near-complete deterrent effects achieved at 900 ppm and sustained through higher concentrations. *Ae. albopictus* exhibited slightly higher repellency of 60% at lower concentration of 300 ppm compared to *Ae. aegypti* which showed 47% repellency at the same concentration, suggesting a marginally greater sensitivity. However, both species reached 100% deterrence at concentrations ≥900 ppm, demonstrating the extract’s potent and consistent oviposition deterrent activity.

**Table 4:** Repellent efficacy (mean %) and Oviposition Activity Index of *A. catechu* nut extract in choice test against both *Aedes* species.

*Aedes* sp.	Concentration (ppm)	Repellent efficacy (mean %)	Oviposition Activity Index (mean ± SE)
***Ae. albopictus***	300	60	−0.44 ± 0.05*
	600	87	−0.77 ± 0.01*
	900	100	−1.00 ± 0.00**
	1200	100	−1.00 ± 0.00**
	1400	100	−1.00 ± 0.00**
	1600	100	−1.00 ± 0.00**
	1800	100	−1.00 ± 0.00**
	2000	100	−1.00 ± 0.00**
***Ae. aegypti***	300	47	−0.31 ± 0.03*
	600	93	−0.87 ± 0.06*
	900	100	−1.00 ± 0.00**
	1200	100	−1.00 ± 0.00**
	1400	100	−1.00 ± 0.00**
	1600	100	−1.00 ± 0.00**
	1800	100	−1.00 ± 0.00**
	2000	100	−1.00 ± 0.00**

Negative OAI values indicate oviposition deterrence, whereas positive values indicate attraction. OAI values are expressed as mean ± SE (n = 5 replicates).

Statistical significance was determined using a one-sample t-test against OAI = 0; *p < 0.01. **Concentrations showing 100% repellency, in which OAI values were −1.00 for all replicates, resulting in zero variance and precluding computation of t-statistics.

**Fig 5:**
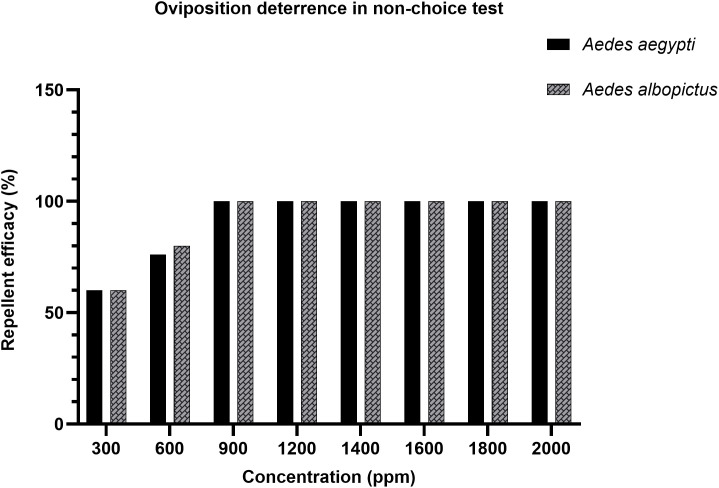
Effective repellency (mean%) in *Ae. aegypti* and *Ae. albopictus* under non-choice test conditions.

Similar patterns were observed in the non-choice test as shown in Table 5. At 300 ppm, both *Ae. aegypti* and *Ae. albopictus* showed 60% deterrence. Repellency increased to 76% and 80%, respectively, at 600 ppm. From 900 ppm onward, effective repellency reached 100% for both species, mirroring the outcomes of the choice test. This is reflected in Fig 5, which shows a progressive increase in repellency up to 900 ppm, after which repellency remained consistently high, indicating maximum deterrent activity. OAI was not calculated for the non-choice assay because mosquitoes were exposed only to treated substrates, in order to assess deterrence under forced conditions. Both species showed complete deterrence from 900 ppm onwards, with *Ae. albopictus* exhibiting slightly higher repellency than *Ae. aegypti* at lower concentrations.

**Table 5:** Repellent efficacy (mean %) of *A. catechu* nut extract in non-choice test against both *Aedes* species.

Mosquitoes	Control	Concentrations (ppm)							
		300	600	900	1200	1400	1600	1800	2000
***Ae. albopictus***	0	60	80	100	100	100	100	100	100
***Ae. aegypti***	0	60	76	100	100	100	100	100	100

Values represent the percentage of egg-laying deterrence at each concentration.

**Fig 6:**
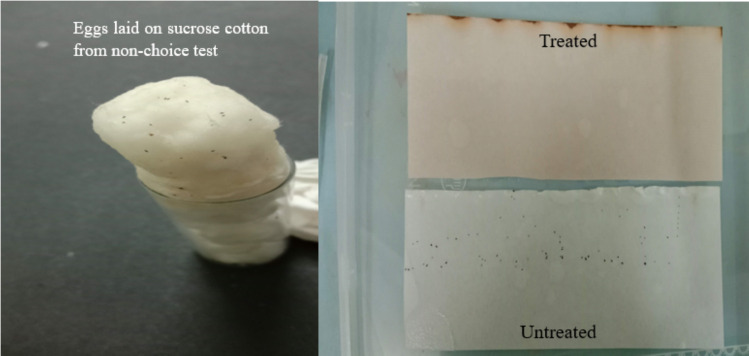
Oviposition behaviour of *Aedes* species in choice and non-choice tests using treated substrates.

Interestingly, behavioural responses under non-choice conditions highlighted the strong aversion of mosquitoes to treated oviposition substrates. As shown in Fig 6, the treated filter paper (top right) shows a complete absence of eggs, indicating strong avoidance of the *A. catechu* nut extract-treated substrate, however, the untreated filter paper (bottom right) is visibly marked with numerous eggs, reflecting normal oviposition activity in the absence of the extract (choice test). In contrast, the left panel displays eggs laid on cotton soaked in sucrose solution, which was originally intended as a food source rather than an oviposition site. This behaviour was observed only in the presence of treated substrates (non-choice test), suggesting that in the absence of a preferred laying surface, mosquitoes redirected their egg-laying activity to the cotton. This behaviour further supports the repellency of the extract and its potential to override oviposition preferences, even under constrained environmental conditions. Together, these results confirm the robust oviposition deterrent properties of *A. catechu* nut extract. Complete deterrence was achieved at concentrations ≥900 ppm in both assay types, indicating that the extract effectively disrupts egg-laying behaviour in *Aedes* mosquitoes.

### LCMS liquid chromatography-mass spectrometry

LC–MS analysis of *A. catechu* nut extract revealed seven distinct peaks, corresponding to seven compounds as presented in the supplementary, S1 file. Of these, three compounds were identified namely, N-Lauryl diethanolamine, arecoline, and arecaidine based on their observed m/z values, calculated molecular weights and spectral information from PubChem (Table 6). Notably, N-Lauryl diethanolamine has previously been reported [26] to exhibit significant antimicrobial and biological activity, including the inhibition of cell proliferation and modulation of cholesterol homeostasis. Additionally, it has been associated with lysosomotropic behaviour, whereby the compound accumulates within lysosomes and may induce cellular stress and apoptosis. Another major compound, arecoline, detected in the extract, is an alkaloid derived from *A. catechu* that has been shown to possess a wide range of biological activities. It has been reported to exert antimicrobial, anti-inflammatory, antiparasitic, and anthelmintic effects. Of particular interest is its acetylcholinesterase (AChE) inhibitory activity, which aligns with the primary mode of action of widely used synthetic insecticides such as organophosphates and carbamates [27]. This suggests a potential neurotoxic mechanism in insect control. Previous study [28] has indicated that, in addition to its pharmacological effects, arecoline can cause growth retardation and developmental abnormalities in animal models, further supporting its potential insecticidal activity. Based on the previous findings [12] arecoline and arecaidine exerted insecticidal property and strong inhibitory effects on carboxylesterase activity against *P. xylostella* larvae, which plays a crucial role in metabolic detoxification processes in insects.

**Table 6:** LC–MS analysis of *A. catechu* nut extract identifying chemical compounds, retention times, and molecular weights.

S/N	RT	Molecular weight	Compound name	Molecular formula	Activity
**1**	3.042	273.2682	N-Lauryldiethanolamine	C_16_H_35_NO_2_	Antimicrobial [26]
**2**	3.299	155.0957	Arecoline	C_8_H_13_NO_2_	Insecticidal, and enzyme inhibition [12,27]
**3**	3.499	141.08	Arecaidine	C_7_H_11_NO_2_	Insecticidal and enzyme inhibition [12]

S/N: Signal notice; RT: Retention time.

Collectively, these findings from previous studies support the insecticidal, enzyme inhibition and antimicrobial patterns of arecoline, arecaidine, and N-lauryldiethanolamine, which were identified through LC-MS analysis of *A. catechu* nut extract. This evidence reinforces the potential utility of the extract as a source of bioactive compounds with insecticidal properties.

## Discussion

The present study provides robust evidence for the insecticidal efficacy of *A. catechu* nut extract against two key mosquito vector species, *Ae. aegypti* and *Ae. albopictus*, exhibiting a wide range of bioactivities including adulticidal, ovicidal, and oviposition deterrent effects. Adulticidal bioassays demonstrated a clear concentration-dependent increase in mortality for both species. Probit regression analysis produced LC₅₀ values of 1201.00 ppm for *Ae. aegypti* and 1214.96 ppm for *Ae. albopictus*, indicating substantial efficacy at moderate concentrations. Regarding ovicidal activity, a strong dose-response pattern was also evident, with complete egg mortality recorded at 1600 ppm for *Ae. aegypti* and at a slightly lower concentration of 1400 ppm for *Ae. albopictus,* suggesting a greater intrinsic susceptibility of the latter species to the ovicidal components of the extract. Moreover, the extract demonstrated pronounced oviposition deterrent activity under both dual-choice and non-choice experimental conditions. The deterrent effect exhibited a strong concentration-dependent trend, with complete suppression of oviposition observed at concentrations ≥900 ppm for both *Aedes* species. Behavioural responses under non-choice conditions further substantiated the extract’s deterrent potential, as indicated by the total absence of eggs on treated substrates and a noticeable diversion of oviposition toward non-preferred or atypical surfaces. Taken together, these findings emphasize the broad-spectrum bioactivity of *A. catechu* nut extract, which effectively disrupts multiple developmental stages and modulates essential reproductive behaviours in *Ae. aegypti* and *Ae. albopictus*.

Complementing the bioassay results, LC-MS analysis of the *A. catechu* nut extract identified several major phytochemicals with recognized bioactivity, namely N-lauryldiethanolamine, arecoline, and arecaidine. Arecoline, the principal alkaloid of *A. catechu*, exhibits notable insecticidal and physiological activity across various pest species. Recent study [29] has demonstrated that arecoline exposure induces significant intestinal metabolic dysfunction in *Spodoptera litura* larvae, a major agricultural pest. Specifically, arecoline was found to suppress the activity of critical digestive enzymes, acetyl CoA carboxylase, lipase, α-amylase, and trypsin, leading to impaired digestion and nutrient assimilation. Additionally, transcriptomic analysis revealed the downregulation of genes involved in lipid metabolism suggesting disruption of energy metabolism as a key mode of toxicity. This enzymatic suppression is accompanied by oxidative stress modulation, indicated by increased superoxide dismutase activity and reduced malondialdehyde levels, reflecting arecoline’s systemic physiological impact on larval development and survival.

Moreover, arecoline has shown broad-spectrum bioactivity in structural derivative studies. Modified arecoline compounds have demonstrated potent antiviral and antifungal properties. These derivatives exhibit significant disruption of viral coat proteins (e.g., in tobacco mosaic virus), causing fragmentation and inactivation, as confirmed through transmission electron microscopy and molecular docking. This indicates that arecoline and its analogues may interact with protein structures critical to pathogen or pest viability, suggesting a mechanism involving protein binding and interference with structural integrity [30]. Traditionally, arecoline has also been recognized for its antiparasitic and insect-expelling properties, supporting its role in pest management. However, it is worth noting that while arecoline is a potent bioactive agent, it also exhibits cytotoxic effects, which underscores the importance of dosage control and formulation in potential insecticidal applications [31]. Importantly, arecoline itself exhibited acetylcholinesterase (AChE) inhibitory activity, analogous to organophosphates and carbamates, classic neurotoxic insecticides, suggesting that arecoline may act via cholinergic disruption in insect nervous systems [27]. Beyond its role as a muscarinic receptor agonist, arecoline has been associated with several adverse effects, including growth retardation and developmental abnormalities in animal embryos such as zebrafish, chickens, and mice [28].

Complementing these findings, arecaidine, another principal alkaloid derived from *A. catechu*-exerts its insecticidal activity through distinct biochemical mechanisms. Notably, it inhibits acetylcholinesterase (AChE), resulting in the accumulation of acetylcholine at synaptic junctions and subsequent disruption of neural transmission, a neurotoxic mechanism commonly associated with organophosphate and carbamate insecticides. Additionally, arecaidine has been reported to inhibit carboxylesterase activity, thereby impairing the insect’s detoxification pathways and enhancing its overall toxic effect [12]

*N*-lauryldiethanolamine, a surfactant-like compound identified in the *A. catechu* nut extract, is also likely to contribute to its insecticidal activity. While this compound is predominantly employed in industrial applications such as emulsifiers and antistatic agents, emerging evidence points to its notable cytotoxic potential. In a recent study [26], *N*-lauryldiethanolamine was among a limited group of extractable compounds that exhibited pronounced biological activity in human U2OS cells, as assessed using the Cell Painting Assay. A 61% induction was observed at the lowest tested concentration of 3 µM, indicating a wide-ranging effect on cellular function. Crucially, N-lauryldiethanolamine significantly reduced cell viability even at low concentrations, suggesting that its mechanism may involve membrane disruption or the induction of cellular stress responses. These properties are particularly relevant in the context of insecticidal activity, as many insecticides act by compromising cell membrane integrity or inducing systemic cytotoxicity. Although its specific activity in insects has not yet been characterized, the compound’s potent and reproducible effects in mammalian cells imply that it may play a role in the adulticidal or ovicidal action observed in *A. catechu* extract. Given its amphiphilic molecular structure and its ability to disturb cellular systems, N-lauryldiethanolamine may act synergistically with other bioactive constituents such as arecoline and arecaidine, contributing to the extract’s overall insecticidal profile.

In line with these findings, several botanical investigations have focused on plant-derived compounds as alternative strategies for mosquito control, reflecting the growing interest in natural insecticides. For instance, it was reported [32] that confertifolin, a bioactive compound extracted from *Polygonum hydropiper*, exhibited potent larvicidal, adulticidal, ovicidal, and repellent activities against *An. stephensi* and *Cx. quinquefasciatus*. A subsequent study by the same authors further demonstrated comparable multistage efficacy of confertifolin against *Ae. albopictus*, underscoring its ability to disrupt multiple developmental stages and reinforcing the broad-spectrum entomotoxic potential of *P. hydropiper* [33].

Similarly, other findings showed that silver nanoparticles (AgNPs) synthesized from *Vernonia anthelmintica* seed extract exhibited strong larvicidal activity against *Ae. aegypti* and *Cx. quinquefasciatus*. The insecticidal mechanism involved elevated activity of detoxification enzymes, including α- and β-carboxylesterases and glutathione S-transferase, indicating metabolic disruption. Additionally, notable histopathological damage to the midgut epithelium and peritrophic membrane was observed, suggesting a dual mode of action involving both biochemical and cellular impairment [34]. Moreover, another study revealed that emodin from *Aspergillus terreus* showed good larvicidal activity against *Ae. aegypti*, *Cx. quinquefasciatus and An. stephensi and* inhibited key detoxification enzymes such as acetylcholinesterase, α and β carboxylesterases, and phosphatases in *Ae. aegypti*, *An. stephensi*, and *Cx. quinquefasciatus*. The study also reported tissue damage in the midgut, hindgut, and nerve ganglia [35]. Expanding on the diversity of natural insecticidal sources, marine-derived compounds have also shown considering activity. The larvicidal efficacy of methanolic extract from the red seaweed *Gracilaria corticata* against three major mosquito vectors: *Ae. aegypti*, *An. stephensi*, and *Cx. quinquefasciatus* was also studied [36]. The extract exhibited significant larvicidal effects across all species. Importantly, biochemical assays confirmed that acetylcholinesterase (AChE) activity was significantly inhibited in all three, suggesting a consistent neurotoxic mode of action.

In addition to marine-derived compounds, terrestrial aquatic plants such as *Azolla pinnata* have also shown notable insecticidal properties with reported LC₅₀ values of 2572.45 ppm for *Ae. aegypti* and 2329.34 ppm for *Ae. albopictus* [22]. While such plant-derived agents are valuable, previous research has predominantly focused on leaves, whole plants, or nanoparticles derived from seed extract. In contrast, the present study evaluates the insecticidal efficacy of *A. catechu* nut extract, thereby contributing meaningful insight into the bioactivity of the seed itself, an underexplored plant part in mosquito control. This nut-specific approach broadens the phytochemical scope of botanical insecticides. Furthermore, while earlier study on *A. catechu* primarily focused on larvicidal activity [10], the present investigation expands this scope by evaluating its efficacy across multiple developmental stages, including adulticidal, ovicidal, larvicidal, and oviposition deterrence effects against *Ae. aegypti* and *Ae. albopictus*.

This comprehensive assessment offers a clearer understanding of the insecticidal properties of *A. catechu* nut extract and reinforces its potential as a viable candidate for inclusion in botanical-based mosquito vector control programs. Importantly, the findings also emphasize the value of extending entomological investigations beyond traditionally studied plant tissues to include underexplored components such as seeds and nuts, which, as evidenced here, can offer promising and chemically diverse alternatives for effective mosquito vector control.

## Conclusion

This study demonstrated the insecticidal efficacy of *A. catechu* nut extract against *Ae. aegypti* and *Ae. albopictus* across multiple bioassays. In adulticidal tests, the extract exhibited a dose-dependent increase in mortality, with LC₅₀ values of 767.501 ppm for *Ae. aegypti* and 758.278 ppm for *Ae. albopictus*. In ovicidal assays, *Ae. aegypti* and *Ae. albopictus* showed 45% and 50% egg mortality at 300 ppm, respectively, with complete mortality observed at 1600 ppm for *Ae. aegypti* and 1400 ppm for *Ae. albopictus*. Oviposition deterrent tests revealed 47% and 60% repellency at 300 ppm in *Ae. aegypti* and *Ae. albopictus*, respectively, with complete deterrence (100%) achieved at ≥900 ppm under choice test. Whereas, under non-choice conditions, 60% deterrence was achieved at 300 ppm with complete deterrence (100%) achieved at 900 concentrations and above for both *Aedes* species. LC–MS analysis identified arecoline, arecaidine, and N-lauryldiethanolamine as major bioactive constituents, suggesting neurotoxic and enzyme-inhibitory mechanisms of action. These results confirm the potential of *A. catechu* nut extract as an effective, plant-based bioinsecticide for integrated *Aedes* mosquito control.

## Supporting information

S1 File LC-MS/MS compound identification report of methanolic Soxhlet extract. (PDF)
