# Supplementary material for: Insecticidal potential of Areca catechu nut extract against multiple life stages of Aedes aegypti and Aedes albopictus
Source: PLoS One. 2026 Feb 2;21(2):e0341897. doi: 10.1371/journal.pone.0341897 (PMC12863473; doi:10.1371/journal.pone.0341897)
Supplement: S1 File — (PDF) [file pone.0341897.s001.pdf]

Qualitative Compound Report

**Data File**	methanol (sox).d	**Sample Name**	methanol (sox)
**Sample Type**	Sample	**Position**	P1-A3
**Instrument Name**	Instrument 1	**User Name**	
**Acq Method**	MADHURI.m	**Acquired Time**	06-Apr-21 5:57:10 PM
**IRM Calibration Status**	Success	**DA Method**	test.m
**Comment**			

**Sample Group**		**Info.**
**Acquisition SW**	6200 series TOF/6500 series	
**Version**	Q-TOF B.05.01 (B5125.1)	

Fragmentor Voltage 175 **Collision Energy** 0 **Ionization Mode** ESI

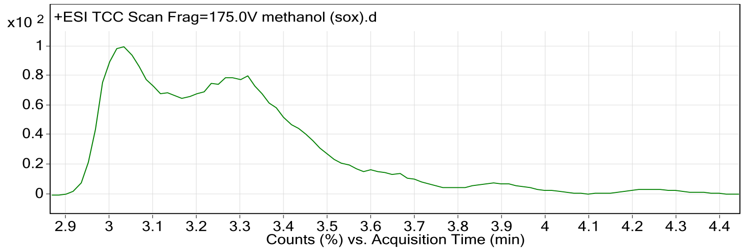

Fragmentor Voltage 175 **Collision Energy** 0 **Ionization Mode** ESI

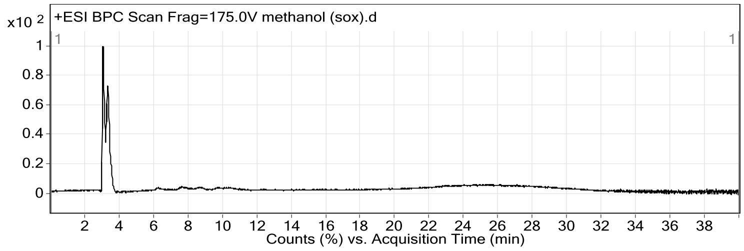

Compound Table

Compound Label	RT	Mass	Name	Formula	MFG Formula	MFG Diff (ppm)	DB Formula	DB Diff (ppm)
Cpd 1: C16 H35 N O2	3.042	273.2682		C16 H35 N O2	C16 H35 N O2	-5.06	C16 H35 N O2	
Cpd 2: C8 H13 N O2	3.299	155.0957		C8 H13 N O2	C8 H13 N O2	-6.98	C8 H13 N O2	
Cpd 3: C7 H11 N O2	3.449	141.08		C7 H11 N O2	C7 H11 N O2	-7.5	C7 H11 N O2	
Cpd 4: D-(+)-Cellobiose	3.637	342.1163	D-(+)-Cellobiose	C12 H22 O11	C12 H22 O11		C12 H22 O11	-0.23
Cpd 5: C16 H35 N O3	3.666	289.2616		C16 H35 N O3	C16 H35 N O3	0.16	C16 H35 N O3	
Cpd 6: C36 H66 N6 O6	3.901	678.5042		C36 H66 N6 O6	C36 H66 N6 O6	0.21	C36 H66 N6 O6	
Cpd 7: C11 H18 N4	4.249	206.1532		C11 H18 N4	C11 H18 N4	-0.45	C11 H18 N4	

Compound Label	*m/z*	RT	Algorithm	Mass
Cpd 1: C16 H35 N O2	274.2755	3.042	Find by Molecular Feature	273.2682

Compound Chromatograms

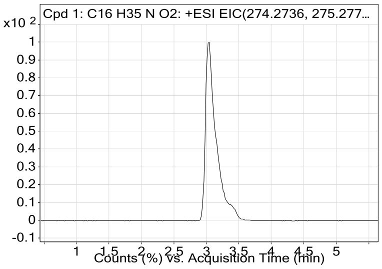

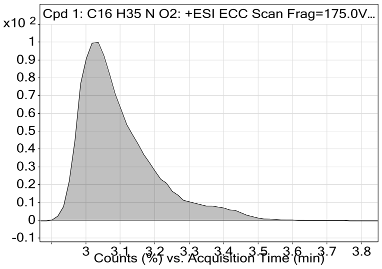

MFE MS Spectrum

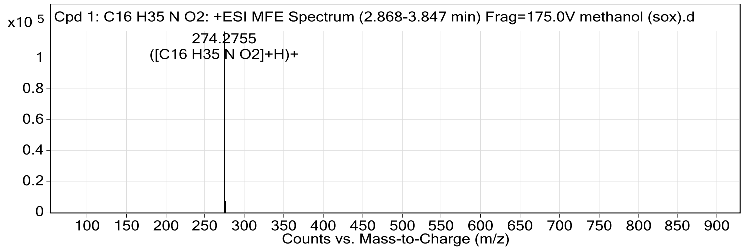

MFE MS Zoomed Spectrum

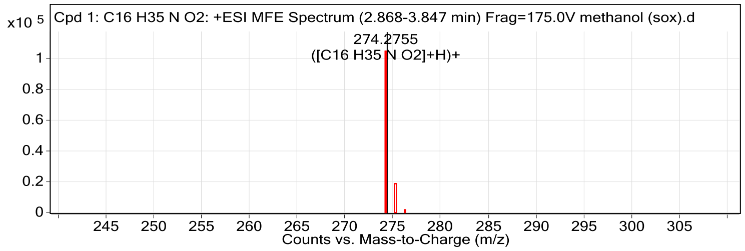

MS Spectrum Peak List

m/z	z	Abund	Formula	Ion
274.2755	1	117430.87	C16 H35 N O2	(M+H)+
275.2785	1	8011.03	C16 H35 N O2	(M+H)+
276.2812	1	301.3	C16 H35 N O2	(M+H)+

Predicted Isotope Match Table

Isotope	*m/z*	Calc m/z	Diff (ppm)	Abund %	Calc Abund %	Abund Sum %	Calc Abund Sum %
1	274.2755	274.2741	-5.11	100	100	93.39	83.25
2	275.2785	275.2774	-4.07	6.82	18.16	6.37	15.12
3	276.2812	276.2802	-3.75	0.26	1.97	0.24	1.64

Compound Label	*m/z*	RT	Algorithm	Mass
Cpd 2: C8 H13 N O2	156.103	3.299	Find by Molecular Feature	155.0957

Compound Chromatograms

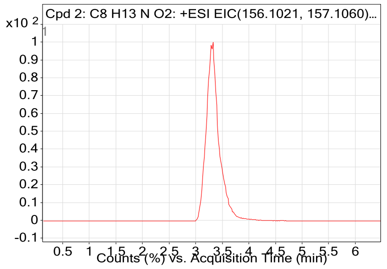

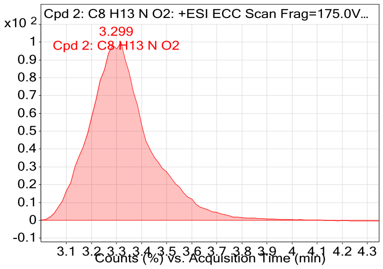

MFE MS Spectrum

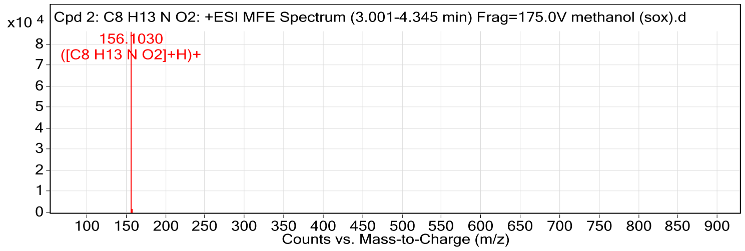

MFE MS Zoomed Spectrum

Qualitative Compound Report

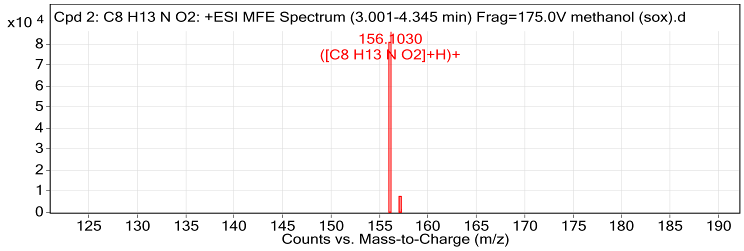

MS Spectrum Peak List

m/z	z	Abund	Formula	Ion
156.103	1	86111.74	C8 H13 N O2	(M+H)+
157.1067	1	2221.76	C8 H13 N O2	(M+H)+

Predicted Isotope Match Table

Isotope	*m/z*	Calc m/z	Diff (ppm)	Abund %	Calc Abund %	Abund Sum %	Calc Abund Sum %
1	156.103	156.1019	-6.84	100	100	97.48	91.53
2	157.1067	157.1051	-10.6	2.58	9.26	2.52	8.47

Compound Label	*m/z*	RT	Algorithm	Mass
Cpd 3: C7 H11 N O2	142.0873	3.449	Find by Molecular Feature	141.08

Compound Chromatograms

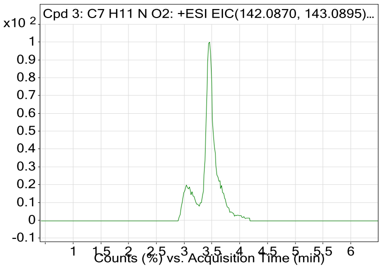

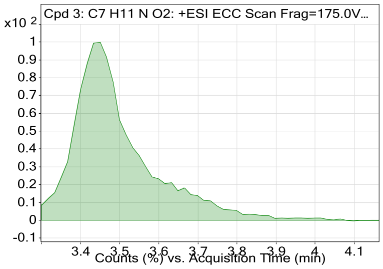

MFE MS Spectrum

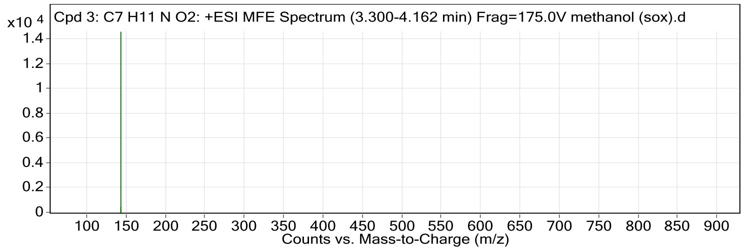

MFE MS Zoomed Spectrum

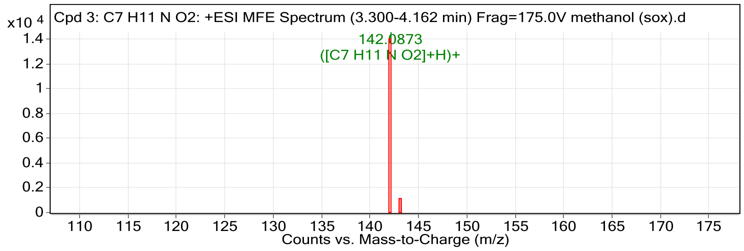

MS Spectrum Peak List

m/z	z	Abund	Formula	Ion
142.0873	1	14627.74	C7 H11 N O2	(M+H)+
143.0898	1	525.08	C7 H11 N O2	(M+H)+

Predicted Isotope Match Table

Isotope	*m/z*	Calc m/z	Diff (ppm)	Abund %	Calc Abund %	Abund Sum %	Calc Abund Sum %
1	142.0873	142.0863	-7.61	100	100	96.53	92.46
2	143.0898	143.0894	-2.99	3.59	8.15	3.47	7.54

Compound Label	Name	*m/z*	RT	Algorithm	Mass

Cpd 4: D-(+)-Cellobiose	**D-(+)-Cellobiose**	365.1055	3.637	Find by Molecular Feature	342.1163

Compound Chromatograms

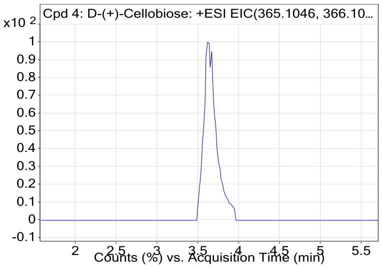

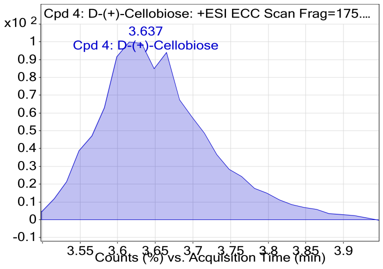

MFE MS Spectrum

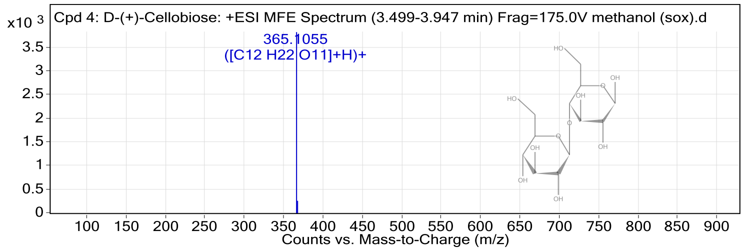

MFE MS Zoomed Spectrum

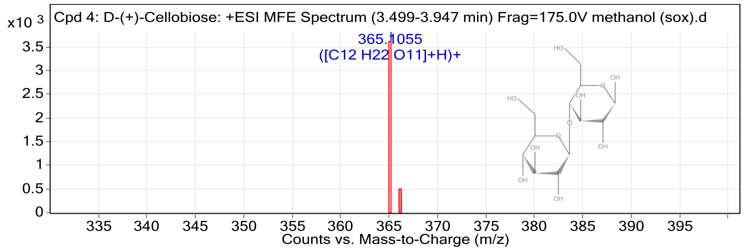

MS Spectrum Peak List

m/z	z	Abund	Formula	Ion
365.1055	1	3825.78	C12 H22 O11	(M+H)+
366.1091	1	281.8	C12 H22 O11	(M+H)+

Predicted Isotope Match Table

Isotope	*m/z*	Calc m/z	Diff (ppm)	Abund %	Calc Abund %	Abund Sum %	Calc Abund Sum %
1	365.1055	365.1052	-0.96	100	100	93.14	88.07
2	366.1091	366.1075	-4.2	7.37	13.55	6.86	11.93

Compound Structure

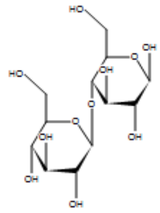

Compound Label	*m/z*	RT	Algorithm	Mass
Cpd 5: C16 H35 N O3	290.269	3.666	Find by Molecular Feature	289.2616

Compound Chromatograms

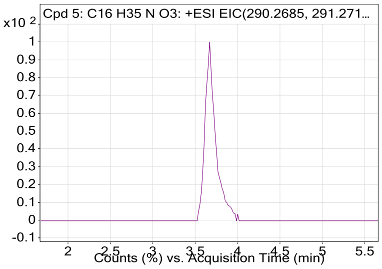

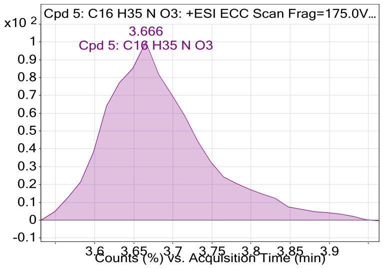

MFE MS Spectrum

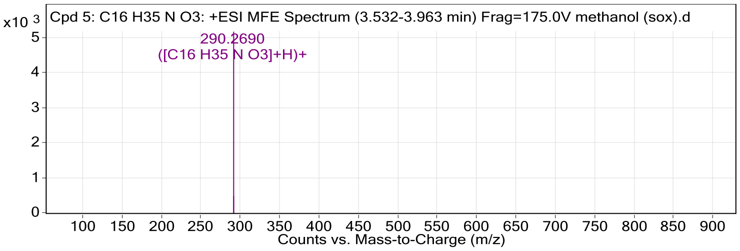

MFE MS Zoomed Spectrum

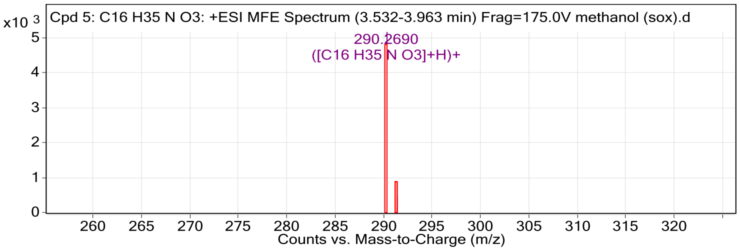

MS Spectrum Peak List

m/z	z	Abund	Formula	Ion
290.269	1	5180.78	C16 H35 N O3	(M+H)+
291.2718	1	494.77	C16 H35 N O3	(M+H)+

Predicted Isotope Match Table

Isotope	*m/z*	Calc m/z	Diff (ppm)	Abund %	Calc Abund %	Abund Sum %	Calc Abund Sum %
1	290.269	290.269	0.02	100	100	91.28	84.6
2	291.2718	291.2723	1.61	9.55	18.2	8.72	15.4

Compound Label	*m/z*	RT	Algorithm	Mass
Cpd 6: C36 H66 N6 O6	701.4936	3.901	Find by Molecular Feature	678.5042

Compound Chromatograms

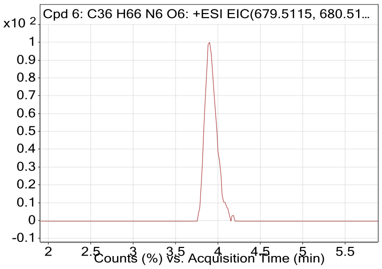

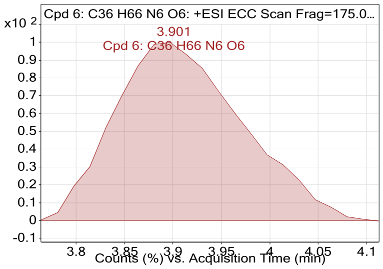

MFE MS Spectrum

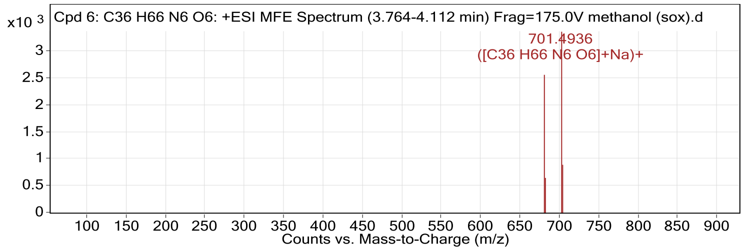

MFE MS Zoomed Spectrum

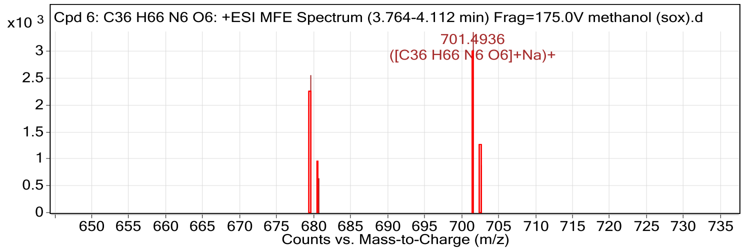

MS Spectrum Peak List

m/z	z	Abund	Formula	Ion
679.5115	1	2558.96	C36 H66 N6 O6	(M+H)+
680.514	1	656.7	C36 H66 N6 O6	(M+H)+
701.4936	1	3364.45	C36 H66 N6 O6	(M+Na)+
702.4962	1	896.97	C36 H66 N6 O6	(M+Na)+

Predicted Isotope Match Table

Isotope	*m/z*	Calc m/z	Diff (ppm)	Abund %	Calc Abund %	Abund Sum %	Calc Abund Sum %
1	679.5115	679.5117	0.25	100	100	79.58	70.36
2	680.514	680.5147	1.07	25.66	42.13	20.42	29.64

Compound Label	*m/z*	RT	Algorithm	Mass
Cpd 7: C11 H18 N4	229.1425	4.249	Find by Molecular Feature	206.1532

Compound Chromatograms

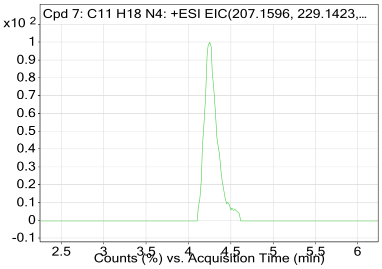

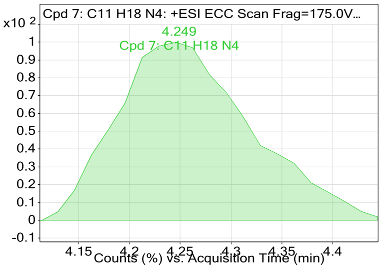

MFE MS Spectrum

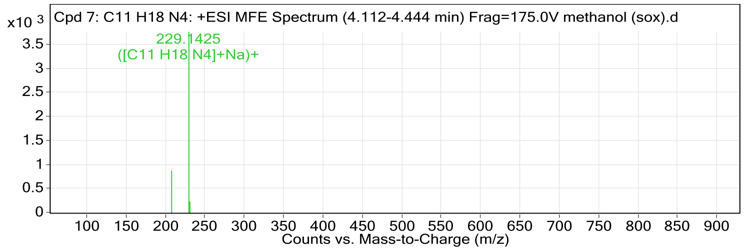

MFE MS Zoomed Spectrum

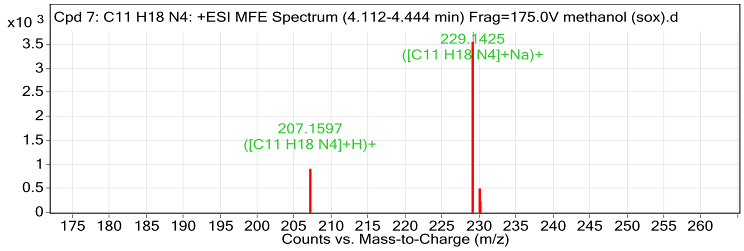

MS Spectrum Peak List

m/z	z	Abund	Formula	Ion
207.1597	1	893.56	C11 H18 N4	(M+H)+
229.1425	1	3769.35	C11 H18 N4	(M+Na)+
230.1452	1	247.41	C11 H18 N4	(M+Na)+

Predicted Isotope Match Table

Isotope	*m/z*	Calc m/z	Diff (ppm)	Abund %	Calc Abund %	Abund Sum %	Calc Abund Sum %
1	207.1597	207.1604	3.26	100	100	100	100

--- End Of Report ---
